# Editorial: Impaired bone healing due to bone disuse and osteometabolic disorders

**DOI:** 10.3389/fendo.2024.1395485

**Published:** 2024-03-28

**Authors:** Ariane Zamarioli, Melissa A. Kacena, José B. Volpon

**Affiliations:** ^1^ Department of Orthopaedics and Anaesthesiology, Ribeirão Preto Medical School, University of São Paulo, Ribeirao Preto, Brazil; ^2^ Department of Orthopaedic Surgery, Indiana University School of Medicine, Indianapolis, IN, United States; ^3^ Richard L. Roudebush VA Medical Center, Indianapolis, IN, United States; ^4^ Indiana Center for Musculoskeletal Health, School of Medicine, Indiana University, Indianapolis, IN, United States

**Keywords:** osteoporosis, bone fracture, bone healing, bone disease, osteometabolic disease

Fracture healing involves an intricate physiological repair process, during which bone formation occurs through a sequence of events, encompassing inflammation, callus formation, and calcification, followed by bone remodeling until its mechanical and biological aspects are restored. The process of fracture healing is intricately regulated by various cellular responses, each orchestrated by specific intracellular signaling pathways. Most fractures will spontaneously heal within six to eight weeks. Although this process involves a well-coordinated sequence of biological events, some fractures will not heal spontaneously and may lead to delayed healing or non-union. A fracture repair is considered delayed when it takes longer than expected to heal, depending on the patient’s age, type of fracture, and fixation method. Fracture non-union occurs when a fracture fails to heal spontaneously within nine months, with no signs of bone healing for three consecutive months. In developed countries, approximately one out of 100 individuals sustain a bone fracture annually, and within this population, non-union occurs in 5%-10% of cases. Aside from the medical complications due to delayed or non-union healing (i.e., pain, fibrotic adherence, limited range of motion, deformity), compromised fracture healing adversely affects life quality and imposes significant financial costs globally. Many factors are known to disrupt the typical progression of fracture healing signaling paths.

While aging is a predominant factor contributing to disrupted fracture healing, various further disorders and situations can also elevate the chances of healing disorders regardless of age. Individuals with conditions such as diabetes, anemia, or infection face an increased likelihood of nonunion following fractures. The mechanisms and features underlying bone fracture failure differ based on the pathogenesis of the primary metabolic dysfunction. Disuse plays a pivotal role in the pathogenesis of disrupted bone healing in numerous neurological disorders impairing mobility, including cerebral palsy, stroke, and spinal cord injury, as well as during prolonged bed rest, immobilization, or exposure to microgravity.

This Topic, “Impaired bone healing due to bone disuse and osteometabolic disorders”, offers an overview of the current therapeutic approaches, along with an insight into the pathogenesis and the risk factors for disrupted fracture healing.

The selected articles published in this Research Topic cover various aspects of unbalanced bone metabolism, assembling one original research paper focused on validating a radiographic system to evaluate bone healing in animals; three involved in the identification of biomarkers to predict fracture risk; and three that investigated therapeutic approaches to stimulate bone repair.

Bone healing investigations are often performed in animal studies, providing important knowledge about pathogenesis and allowing for therapeutic approaches to stimulate bone regeneration. Alentado et al. sought to characterize the longitudinal course of the modified Radiographic Union Score for Tibia fractures (mRUST), which is a validated method to assess fracture union in humans by means of X-ray images. The authors further aimed to establish a comparison between mRUST and another conventional fracture assessment method in rodents. Using this validated longitudinal bone healing measurement could substantially reduce costs and animal usage in studies. The authors evaluated the fracture repair process in mice at timepoints following the injury by microcomputed tomography (μCT), histomorphometry, and mechanical tests. Three orthopedic surgeons conducted mRUST scoring of plain X-ray images in a randomized, blinded manner. The authors reported that mRUST scores exhibited strong correlations with biomechanical, histomorphometry, and μCT parameters, thus being a reliable measure of murine fracture healing, with the advantage of being more cost-effective and non-terminal.

In a cross-sectional study with 900 subjects, Xu et al. reported that elevated serum undercarboxylated osteocalcin (ucOC) levels were linked to reduced bone mass and a higher incidence of osteopenia and osteoporosis among the Chinese population, affecting the lumbar spine, femoral neck, and total hip. Undercarboxylated osteocalcin (ucOC) is a form of osteocalcin that lacks full carboxylation and is involved in bone homeostasis, glucose regulation, energy density, bone turnover markers, and population health. Additionally, higher ucOC levels were associated with increased levels of P1NP and β-CTX, and an elevated risk of osteopenia or osteoporosis in both genders.

As mentioned before, bone disuse is a known risk factor for bone loss and healing disorders. Spinal cord injury is known to induce rapid and intense bone loss, which may lead to a higher risk of osteoporotic fracture. The identification of a biomarker to predict fracture risk in this population remains under investigation. Lincoln et al. aimed to explore microRNA-mediated mechanisms associated with bone loss in SCI individuals. After running a transcriptomic study to assess microRNA expression, the authors selected miR-148a-3p for an additional investigation, in which they detected an upregulation in acute SCI individuals vs chronic SCI, as well as in acute injury vs no injury. The authors also performed CT scans on all subjects and detected greater levels of marrow adiposity in individuals with chronic SCI compared to acute. Therefore, the authors reported that levels of miR-148a-3p exhibited a negative correlation with distal femoral diaphysis marrow adiposity and suggested this microRNA as a factor contributing to osteoporosis after spinal cord injury and a prospective therapeutic target in the future.

Liu et al. conducted a genomic study to uncover susceptibility modules and hub genes linked to Diabetes mellitus and bone healing. Based on a weighted co-expression network analysis method, gene expression, and correlation analysis, the authors identified ANXA3 as a biomarker associated with neutrophils in bone healing events by single-cell RNA sequencing assessment. The authors conducted a clinical study to evaluate the expression of ANXA3 using qRT-PCR in individuals with type 2 diabetes and bone non-union. Their findings indicated that the increased expression of ANXA3 might contribute to bone non-union in diabetic patients by mediating neutrophils.

In another study involving diabetes and disruption in fracture healing, Campos et al. reported a 17% decrease in bone mass and microstructural parameters associated with bone formation in diabetic rats when compared to control animals, which induced a severe impairment in fracture healing. Accordingly, the authors detected a significant downregulation in genes linked to osteoblastogenesis (Runx2, Col1a1, Osx), and a 92% decrease in the level of serum insulin-like growth factor I (IGF-1) in diabetic rats versus the controls. Conversely, osteoclastogenesis was increased in diabetic rats when compared to the controls, as the authors reported a 20% higher porosity at the callus, a 33% increase in trabecular separation, and a 318% increase in the levels of serum C terminal telopeptide of type 1 collagen. The authors also addressed the effects of vibration therapy as an effective approach to mitigate the negative effects of diabetes on bone repair. Of note, the authors reported a partial recovery in the serum levels of IGF-1 and RANKL, which stimulated bone formation, allowing adequate fracture repair in diabetic rats that underwent vibration therapy.

Another approach to stimulated bone regeneration was studied by Zamarioli et al. The authors conducted an experimental study in mice sustaining a bone fracture in the lack of gravity, in spaceflight. The authors detected that a single dose of BMP2 locally applied at the femoral fracture gap appears to induce systemic changes on distant bones. Interestingly, the authors identified better outcomes in the weight-bearing bones when treatment was performed on Earth and in bones subjected to higher muscle contraction when treatment was performed in spaceflight. The authors speculated that BMP-2 treatment may work through a pathway involving mechanical loading.

Perini et al. also investigated a therapeutical approach to stimulate bone regeneration. The authors conducted a comparative analysis of neonatal lung and bone marrow-derived endothelial cells (LECs and BMECs, respectively) *in vitro*. To assess the functionality of neonatal ECs *in vivo*, they induced a bone fracture at the femurs in young and aged mice and embedded a collagen sponge to deliver neonatal BMECs at the fracture location. The authors observed that both neonatal LECs and neonatal BMECs exhibited endothelial cell traits *in vitro*. Additionally, they found that neonatal BMECs significantly enhanced endochondral bone formation in aged rodents, suggesting their potential to enhance aged bone healing.

In conclusion, important advancements have been established in bone science and fracture healing, leading to a wide range of improvements in healing disorders. Nevertheless, delayed callus formation and nonunion are still a burden in orthopedics and place significant financial burdens on healthcare systems. Further studies seeking improved therapies and/or tools to predict healing disorders will contribute to our understanding and developing of novel therapeutic approaches to stimulate bone repair processes.

## Author contributions

AZ: Conceptualization, Formal analysis, Project administration, Writing – original draft, Writing – review & editing. MK: Conceptualization, Formal analysis, Supervision, Writing – review & editing. JV: Supervision, Writing – review & editing.

